# Genome-Wide Association Analysis Identifies Candidate Genes Regulating Seed Number per Silique in *Arabidopsis thaliana*

**DOI:** 10.3390/plants9050585

**Published:** 2020-05-02

**Authors:** Huan-Li Jiang, Jun Hong, Yu-Tong Jiang, Shi-Xia Yu, Yan-Jie Zhang, Jian-Xin Shi, Wen-Hui Lin

**Affiliations:** 1 School of Life Sciences and Biotechnology, The Joint International Research Laboratory of Metabolic & Developmental Sciences, Shanghai Jiao Tong University, Shanghai 200240, China; hlj2017jd@sjtu.edu.cn (H.-L.J.); hongjun0699@sjtu.edu.cn (J.H.); JIANGYUTONG@sjtu.edu.cn (Y.-T.J.); shixiayu@sjtu.edu.cn (S.-X.Y.); ljj307@sjtu.edu.cn (Y.-J.Z.); jianxin.shi@sjtu.edu.cn (J.-X.S.); 2 Joint Center for Single Cell Biology, Shanghai Jiao Tong University, Shanghai 200240, China

**Keywords:** GWAS, silique length, seed number per silique, seed density, *PIN3*

## Abstract

Seed weight and number ultimately determine seed yield. Arabidopsis seed number comprised of silique number and seed number per silique (SNS). Comparing seed development and weight, determinants of seed number remain largely uncharacterized. In this study, taking advantage of 107 available Arabidopsis accessions, genome-wide association analysis (GWAS) was employed to identify the candidate genes regulating SNS. GWAS-based genotype and phenotype association analysis identified 38 most significant SNPs marker sites that were mapped to specific chromosomal positions and allowed us to screen for dozens of candidate genes. One of them (*PIN3*) was selected for functional validation based on gene expression analysis. It is a positive regulator of Arabidopsis SNS. Although silique length of *PIN3* loss of function mutant was not significantly changed, its SNS and seed density (SD) were significantly reduced as compared with the wild type. Notably, *PIN3* overexpression lines driven by a placenta-specific promoter STK exhibited significantly shorter siliques, slightly reduced SNS, but significant increased SD compared with wild type, suggesting that *PIN3* positively regulates SD through inducing ovule primordia initiation regardless of the placenta size. Ovule initiation determines the maximal possibility of SNS, and new genes and mechanism regulating SNS through modulating ovule initiation is worth further investigated.

## 1. Introduction

Seeds are reproductive organs of gymnosperms and angiosperms plants, and are also the main harvest in agricultural crops [1]. Seed yield is a key trait that directly determines crop yield, and increasing seed quantity is an effective way to increase crop yield [2]. Compared with the investigation of seed development and seed weight, the regulatory mechanism of seed quantity has not been systematically studied, especially for dicotyledon crops. Rapeseed and soybean are important dicotyledonous crops, and their harvesting organs are all seeds. Increasing fruit number and the number of seeds of single fruit (siliques and pods) are two ways to increase the total number of rapeseed and soybean seeds [3]. *Arabidopsis thaliana*, a model plant of the Brassicaceae family, is a good material for studying the regulatory mechanism of dicotyledonous seed numbers, in which the seed number is determined by both seed number per silique (SNS) and the silique number [4].

Silique number is determined by fertilized flower number, and the seed number produced by each flower is determined by many factors. Factors, such as floral organ development, ovule identity, ovule primordia initiation, male gametophyte and female gametophyte development, double fertilization, and zygotic development all affect the final SNS, and each of these processes is regulated by different genes and signaling pathways [5,6,7,8,9,10,11,12]. The maximum possibility of SNS depends on ovule number, while the maximum possibility of ovule number depends on the ovule identity and ovule primordia initiation. It is well-known that ovule initiation is regulated by genes regulating floral organ development [13,14,15], hormone signals [16,17,18], and environmental factors [19,20].

The ABC model of Arabidopsis flower organ development [21,22] suggested that the normal differentiation of flower organs is regulated by three types of genes: A, B, and C, and they act on adjacent two rounds of flower organs antagonistically [5]. Further investigations have modified the ABC model to the ABCDE model [6]. Functions of Class D genes and class C genes partially overlap, jointly regulating the ovule development; these genes include *SEEDSTICK* (*STK*), *SHATTERPROOF 1* (*SHP1*), and *SHP2* [23,24]. *STK* is specifically expressed in the placenta and ovules even before ovule primordium initiation [25,26], the marker line pSTK::GUS constructed with the STK promoter can specifically indicate ovule primordia initiation and ovule development. Class E genes act as a protein backbone to assist other types of genes in functioning and affect the development of all floral organs [27,28,29,30].

The abnormal development of floral organs, ovules, male and female gametes, will affect the fertilization and zygote development and lead to a reduction in SNS. There are two different ways for increasing SNS, one is keeping the normal process of development and preventing abortion of ovule and seed; the other is increasing ovule initiation to maximize the possible SNS. The latter one could increase SNS absolutely if the developmental process were normal. In *Arabidopsis*, the ovule initiates from placenta that developed from carpel margin meristem (CMM). The subepidermal tissue of the placenta undergoes anticlinal division to form ovule primordia. A relatively homogeneous cell population then forms three different regions of the ovule along the paraxial-distal axis in order: the ovule, the junction, and the nucleus [31,32]. Several negative regulators of this process have been reported. For example, ovule number is reduced in *seu* mutants [14] and the pistils of the *ant seu* bipod are cracked and no ovule primordia are formed [15], indicating that pistil development and CMM production and maintenance are prerequisites for ovule formation. The process of ovule primordia initiation is affected by the above-mentioned class D genes (such as *STK*, *SHP1*, and *SHP2*) [25,26], plant hormones (such as auxin, cytokinin and brassinosteroids), and some transcription factors (for example ANT, HLL, and AP2) [33,34,35,36,37].

Previous studies have shown that the initiation and number of ovule primordia are greatly affected by the size of the placenta. The flowers and placentae of the *ckx3 ckx5* double mutants are larger than those of wild type, so is ovule number, indicating a possible positive correlation between placenta size and ovule number [16]. In addition, BR signaling pathway is involved in SNS regulation in *Arabidopsis*. In the *bzr1-1D* mutant, a higher SNS is accompanied with a higher level of non-phosphorylated BZR1. However, although the placenta of the *bzr1-1D* is also increased, it does not apply enough space for increased ovule, resulting in more seeds in crowded ovules [3]. This result indicated putative new mechanisms beyond the placenta space-mediated ovule initiation, which is rarely studied at present. Finding genes that enhance ovule initiation without being restricted by placental size is of great significance for increasing SNS, total seed quantity, and total seed yield.

Here we present our results in the identification of loci associated with SNS through a genome-wide association analysis (GWAS) using 107 Arabidopsis ecotypes from different geographic locations. Combined with further genetic analysis, we found that *PIN3* is involved in the positive regulation of ovule primordia initiation. *PIN3*, together with other genes discovered in this study, provides genetic resources for the breeding of high-yield crops in agricultural production.

## 2. Results

### 2.1. Seed Number Per Silique Vary Significantly Among Arabidopsis Accessions

**Figure 1:**
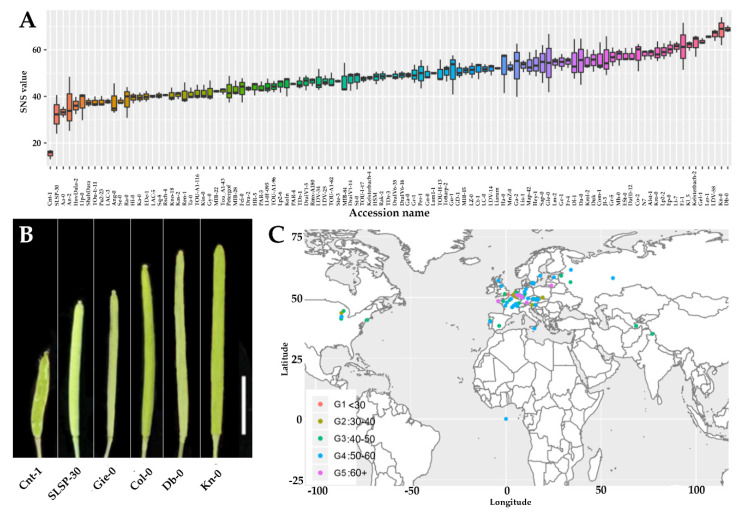
Arabidopsis accessions display significant natural variations in seed number per silique (SNS). (**A**) Boxplot of seed number in 107 Arabidopsis accessions. (**B**) Siliques of low, medium, and high SNS accessions (Bar = 5 mm). (**C**) Geographic distribution of accessions. Groups 1 to 5 correspond to seed number ranking (from low to high).

To identify new genes that regulate seed yield in *Arabidopsis*, we recorded yield relevant phenotypes including silique length, silique number, seed number per silique (SNS), and seed density (SD) in 260 natural accessions of Arabidopsis germplasm with different genetic backgrounds and geographical origins provided by Nottingham Arabidopsis Stock Centre (NASC) [38] (Table S1). Our preliminary results revealed that SNS phenotype was worthy further investigations. Thus, to ensure the accuracy of the data, 153 accessions with delayed flowering, poor growth, and reduced plant vegetative organs were removed. In the remaining 107 accessions, only five well-developed siliques were collected from the 6th to 12th siliques in the main inflorescence axis of each plant used for statistical analysis. Our statistical data revealed that these tested Arabidopsis accessions showed significant variations in SNS when grown under the same condition (Figure 1A). In summary, for all 107 accessions, the SNS exhibited a wide range of 15 to 69, with a mean value of 49. Obvious natural variations in silique length, silique numbers, and SD were also observed (Figure 1B). Notably, SNS was not likely related to their origin locations of those 107 Arabidopsis accessions (Figure 1C).

### 2.2. 38 SNP-SNS Associations Indentified in GWAS

**Figure 2:**
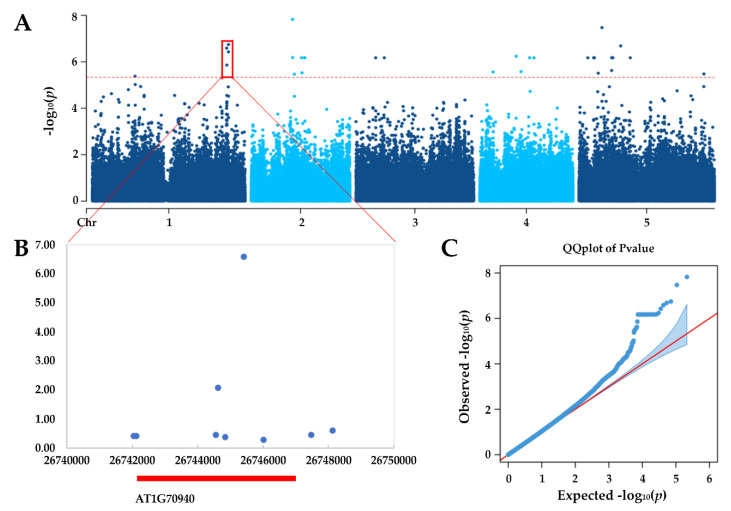
Genome-wide association studies (GWAS) of seed number per silique (SNS) in *Arabidopsis*. (**A**) Manhattan plots of the Fast-LMM model. Red dashed line corresponds to–log10 (*p*-values) > 5.327. (**B**) Local region of significant GWAS signal with predict gene annotation show GWAS signals concentrate upon *PIN3* gene. (**C**) Q-Q plot of the Fast-LMM mode.

In order to identify genetic mechanisms underlying the observed natural variation in SNS, GWAS was used to correlate phenotype and genotype using single nucleotide polymorphisms (SNP) data obtained from public databases [39,40]. GWAS were performed for each SNP using a linear mixed model and the results were analyzed using FaST-LMM [41]. Manhatten and Q-Q plots of GWAS result are illustrated in Figure 2A,C. A significance cutoff *p*-values < 4.7 × 10^−6^ (1/number of markers) detected totally 38 significant SNPs, which were mapped to specific chromosomal positions to screen for dozens of possible candidate genes (Table S2). According to gene expression data from the TAIR database [42], we selected a plausible candidate gene among 38 SNP-trait associations, located besides the most significant GWAS signal in each local region and are highly expressed in flowers and pistils, for further verification (Figure 2B).

### 2.3. PIN3 Is a Positive Regulator of SNS

Among the three putative candidate genes that regulate SNS in *Arabidopsis*, *PIN3* stood out as a priority for further study mainly because of its multiple functions in auxin transport mediated growth and development in plants [43,44]. The in silica data of *PIN3* (Figure S1) [45] implied a putative role for *PIN3* in silique development.

**Figure 3:**
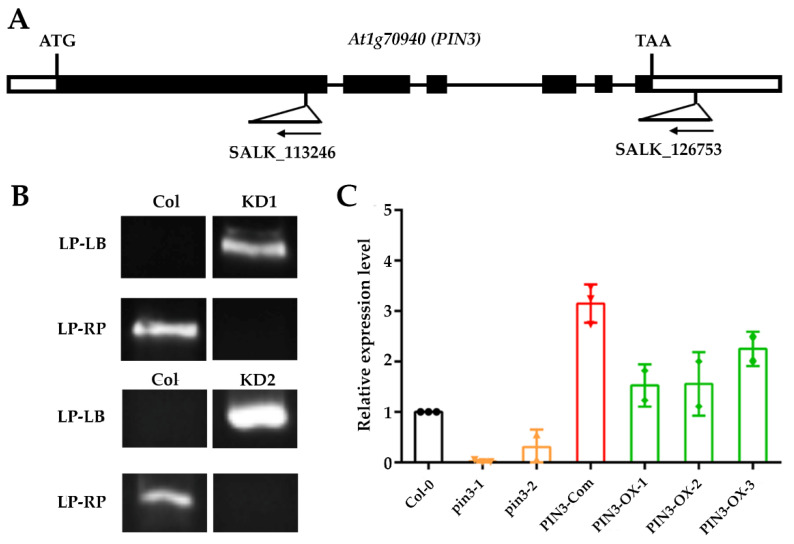
Identification of homozygous T-DNA insertion knockdown (KD) lines and *PIN3* gene expression levels in the inflorescences of different lines. (**A**) Homozygous T-DNA insertion knockdown (KD) lines used in this study. The figure shows location of T-DNA insertion in the gene. The arrow indicates the direction of T-DNA insertion (reverse). (**B**) The left and right genomic primers LP and RP, and the T-DNA border primer LB used for validation of homozygous KD lines. The gel photo shows that the LP-LB primer pair was amplified, while the genomic primer pair LP-RP was not amplified, confirming that the KD mutants was homozygous. (**C**) qRT-PCR of *PIN3* genes in *pin3-1*, *pin3-2*, PIN3-Com (complementation lines), PIN3-OX-1 (overexpression lines), PIN3-OX-2, and PIN3-OX-3 flowers.

**Figure 4:**
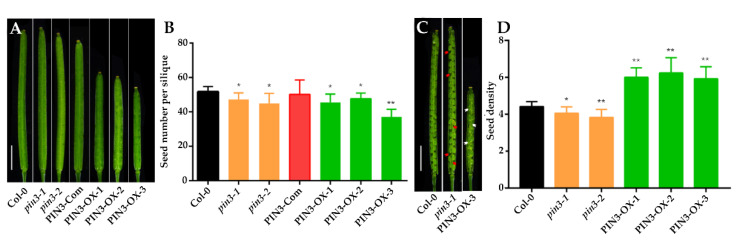
Siliques, seed number per silique (SNS) and seed density (SD) of wild type, *pin3*, PIN3-Com (complementation lines) and PIN3-OX (overexpression lines). (**A**) The silique of Col-0, *pin3-1* and *pin3-2* mutants, PIN3-Com, and PIN3-OXs (Bar = 2mm). (**B**) SNS of Col-0, *pin3-1* and *pin3-2* mutants, PIN3-Com, and PIN3-OXs. (**C**) Dissected siliques of Col-0, *pin3-1* and PIN3-OX-3. Red and white arrows indicate sparsely arranged and crowdedly arranged seed region, respectively (Bar = 2 mm). (**D**) SD of wild type, *pin3* and PIN3-OX. “**” indicates statistical significance at *p*-value < 0.01, “*” indicates statistical significance at *p*-value < 0.05, *p*-value determined by Student’s t-test.

To investigate further the function of the *PIN3* gene in SNS, we searched the SALK homozygous T-DNA insertion mutant library [46] and obtained two homozygous T-DNA insertion lines: SALK_113246 and SALK_126753. The molecular characterization of these two T-DNA mutants at both DNA (Figure 3A,B) and mRNA levels (Figure 3C) verified their homozygous status, in which the expression of *PIN3* gene in both mutants was significantly reduced as compared to wild-type Col-0 (Figure 3C). Therefore, these two mutants were named as *pin3-1* and *pin3-2*, respectively. Silique length in *pin3-1* and *pin3-2* mutants did not differ significantly from that of wild type (Figure 4A), however, SNS in both mutants were significantly lower than that of wild type (Figure 4B). In addition, SDs of the two mutants were also significantly lower than that of wild type, as evidenced by looser and sparser seed arrangement inside the siliques (Figure 4C,D).

We also complemented *pin3-1* or *pin3-2* with a pPIN3::PIN3-GFP construct, and the positive transgenic plants with *pin3-1* background (PIN3-Com, Figure 3C) rescued the SNS and SD phenotypes in *pin3-1* or *pin3-2* (Figure 4B–D). The genetic complementation result indicated that *PIN3* acts as a positive regulator of SNS and SD in *Arabidopsis*.

### 2.4. Overexpression of PIN3 Increases SNS

Because *PIN3* is an auxin polar transporter, the ectopic overexpression of *PIN3* might induce a constitutive response. To detect the specific regulation of *PIN3* in SNS, we transformed Col-0 plants with a PIN3-GUS fusion construct driven by the placental-specific promoter STK. Interestingly, siliques in positive overexpression lines (PIN3-OX, Figure 3C) were shorter than those of Col-0 wild type (Figure 4C). Although *PIN3* overexpression lines exhibited a reduction in the average SNS due to different degrees of abortion (Figure 4D), the overexpression of *PIN3* significantly increased the SD in fully fertilized siliques. Even in siliques with aborted seeds, the SD ratio (of the total number of both aborted and fertile seeds to the length of silique) in *PIN3* overexpression was higher than that of wild type (Figure 4C,D). Furthermore, although silique length was shortened because of unknown reasons, SNS was not decreased proportionally. Importantly, SD in these overexpression mutants increased significantly (Figure 4D).

## 3. Discussion

Seed number and seed weight (Thousand Grain Weight, TGW) is reported to be negatively correlated with seed number in many crops because of limited space and nutrition [47,48], and both seed weight and number contribute to seed yield. Currently there are also reports showing that the seed number and weight are not absolutely negatively correlated [2,3,16], suggesting that increased seed number would be a new way to enhance seed yield if seed weight would not be significantly decreased.

As we mentioned above, the development of Arabidopsis floral organs, ovules, male and female gametes, will affect the fertilization and zygote development. The abnormality in these processes will lead to reduced SNS through adversely influencing fertility and seed set. Previous studies reveal genes negatively regulating SNS through decreasing seed fertility and seed set, including those involved in female gametophyte development [49,50]. Female gametophyte is formed in the nucleus, and the process from macrospore mother cells and functional macrospores to seven-cell and eight-nucleus mature embryo sacs is a continuous process that is generally divided into seven periods of FG1 to FG7 [51,52] with different regulatory genes at each period. For example, development of female gametophytes in the double mutant of *rhf1a rhf2a* stops at the FG1 stage [53]; the development of embryo sac in *prl* mutants stops at the tetranuclear (FG4) stage [54]; and the development of the embryo sac in *nomega* mutants stays in the binuclear (FG2) stage [55,56]. Male gamete development also affects SNS in *Arabidopsis*, which includes two processes: microsporogenesis and male gametogenesis. After mature pollen grains are formed, the male and female gametes complete fertilization in the embryo sac after a process of sperm delivery. These processes are jointly regulated by different genes [57,58,59]. Any abnormality of these genes may indirectly affect SNS finally. Overexpression of genes regulating fertility and seed set does not increase SNS because they do not enhance the ovule initiation and ovule number. Although GWAS analysis of ovule number identified NERD, which positively regulates ovule number and SNS, it still regulated ovule number through helping ovule development and fertility [60]. However, only a few genes were reported to positively regulate SNS mainly through increasing ovule number by elongating placenta size [16]. 

In this study, we used phenotypes of SNS, in a population of natural accessions of Arabidopsis for GWAS analysis. To ensure the accuracy of the data, 153 accessions with delayed flowering, poor growth, and reduced plant vegetative organs were removed and only 107 accessions were further investigated. We identified 38 potential causal loci/genes for SNS regulation. Among the dozens of the genes screened by GWAS, one is *AGM* (*ABNORMAL GAMETOPHYTES*) (Table S2), a known gene that is involved in the regulation of seed set and SNS [61], indicating that additional candidate genes identified through GWAS are likewise involved in SNS regulation.

Here we presented one of the examples, which is not connected to SNS in previous research. One SNP that was located at 26745404 in chromosome 1 with a significant association with SNS in At1g70940, which is also known as *PIN3*, encodes a regulator of auxin efflux. Interestingly, auxin transport has been implied to be associated with ovule development in many plant species such as cucumber, cotton, and rice [18,62,63,64]. In *Arabidopsis*, cytokinin also regulates ovule development through its regulation of *PIN1* and *PIN3,* leading to altered auxin distribution. *PIN3* is reported to be involved in medial tissue development, and its function can be partially complemented by *PIN1* or *PIN7* [65,66]. Thus, *PIN3*, identified by our GWAS study established a potential connection between variation of auxin distribution and ovule initiation in Arabidopsis natural accessions.

The functional characterization of *PIN3* through loss-of-function mutant illustrated that it could play an essential role in SD because there were less seed in mutant silique although the silique length and placenta size have no significant difference between mutant and wild type. The more sparse alignment of seeds in *pin3* siliques indicated the reduced SD in the mutant, which prompted us to investigate whether overexpression of *PIN3* enhances SD. Because *PIN3* is an important auxin polar transporter, the ectopic expression of *PIN3* possibly led to abnormal growth and development, thus, we used a placenta-specific promoter to overexpress *PIN3* in placenta. qRT-PCR results illustrated that the expression level of *PIN3* in PIN3-Com (using native promoter) was higher than Col-0, but PIN3-Com did not have phenotype of higher seed density (SD). According to the prediction (Figure S1) and previous publication, PIN3 showed low expression in placenta (Friml et al. 2002; Ceccato et al. 2013; Chen et al. 2015), indicating that PIN3 increased SD only if it is overexpressed in placenta [66,67,68]. Although both silique length and SNS were reduced, SD ratio was significantly higher in overexpression lines than in wild type, indicating that specifically overexpression of *PIN3* in placenta increases seed number per unit of silique length. In addition, although aborted seeds were observed in some *PIN3* overexpression siliques, there was no significant difference in silique length between siliques with lower or higher abortion ratio, indicating the observed silique length reduction in *PIN3* overexpression lines is not the consequence of seed abortion. Taken together, these results indicated that *PIN3* positive regulates ovule initiation and ovule number without increasing placenta size. 

There are two ways to increase SNS in *Arabidopsis*. One is to increase silique length and the other to increase SD by promoting ovule initiation and ovule number. Compared with increasing the silique length, increasing SD is worth more investigation. Previous studies identified many genes, such as *BZR1*, *CKX3* and *CKX5*, that regulate SNS through their functions on silique length and ovule initiation [3,16]. Different from those genes, *PIN3* regulates SNS through increasing SD by promoting ovule initiation without increasing placenta size. The shortened silique could be the consequence of altered auxin distribution in the placenta tissue in these mutants caused for altered *PIN3* activity. Although pSTK-PIN3 caused problems of shortened placenta and aborted seed setting by unknown reasons, the functional study of *PIN3* in this study revealed a potential new regulatory mechanism for increasing ovule initiation and SNS, which is worth further investigation through a systematic approach.

## 4. Materials and Methods

### 4.1. Plant Material and Growth Conditions

The 107 Arabidopsis thaliana accessions used in this study were kindly shared from the Chao laboratory [69], and the relevant information is listed in Table S1. Arabidopsis T-DNA insertion mutants *pin3-1* (SALK_113246) and *pin3-2* (SALK_126753) were ordered from the SALK Institute (Salk Institute for Biological Studies). We used the Col ecotype as a wild-type control for the above mutants.

Many of the Arabidopsis accessions require vernalization for flowering [70], so we chose to vernalize all different Arabidopsis ecotypes in this study for 3–28 days at 4 °C. After vernalization, they were grown in a light incubator (constant temperature 22 °C, light 16 h, dark 8 h). T-DNA insertion mutants and Col seeds were sterilized, plated on 1/2 MS medium, vernalized for 2–3 days, and transferred to the above-mentioned light incubator for cultivation. After one week, the seedlings were transplanted into the soil (22 °C greenhouse, 16 h light, 8 h dark). The seeds of the transgenic lines were sterilized and seeded on 1/2 MS medium containing 20 mg /L hygromycin to screen transgenic seedlings, which were then transplanted into soil and grown under the above-mentioned long-day conditions.

### 4.2. Phenotypic Analysis of Seed Number

In order to count the average silique number of 107 Arabidopsis ecotypes, we adopt a more convenient statistical method. Three plants of each ecotype were taken. After the siliques matured, the first five incompletely developed siliques of the main inflorescence axis of the *Arabidopsis thaliana* were removed. Five siliques were counted. The mature seeds were tiled on A4 paper, photographed with a digital camera (Canon 600D), and counted using ImageJ software. In the calculation of seed density, the above method was still used to obtain the material. The length of the caryopsis was measured with ImageJ software, and then the carpel wall was removed with a dissecting needle. The total number of seeds (including unfertilized and aborted ones) was measured with the aid of a stereo microscope (Leica DFC290).

### 4.3. Genome-Wide Association Study

The average trait value for each accession was used for the GWAS in this study. 214,051 single nucleotide polymorphisms (SNPs) from 107 accessions were obtained from the 1001 Genomes Project website for *Arabidopsis thaliana*. A GWAS was conducted using a mixed linear model method in Fast-LMM software using the default parameters. The population structure was controlled using the relatedness matrix generated from Fast-LMM. The P-values < 4.7 × 10^−6^ (1/number of markers) were considered significant.

### 4.4. Vector Construction and Transformation Processes of Overexpression and Complementation Lines

The STK promoter sequence was amplified using primer pairs STK XBal-F’/R’ together with KOD high-fidelity DNA polymerase (TOYOBO). Candidate genes *PIN3* sequences: Using the inflorescence cDNA as a template, using primer pairs PIN3- F’/R’, amplification was established by KOD enzyme. The STK promoter sequence was ligated into the pBI101.3 vector, and the amplified candidate gene was inserted into the pSTK-GUS vector to obtain pSTK::PIN3-GUS (pBI101.3) vector.

Vector construction of complementation lines: The *PIN3* candidate gene (including its own promoter) was amplified using primer pairs PIN3C-F’/R’ together with KOD enzyme. The candidate gene was ligated into a pHB vector to obtain pPIN3::PIN3-GFP (pHB) vector.

The pSTK::PIN3-GUS (pBI101.3) vector was transformed into Col-0 plants and placed in the pistil. Transgenic plants with specific high expression of *PIN3* were obtained. The pPIN3::PIN3-GFP (pHB) vector was transformed into *pin3* mutant plants to obtain mutant complementation lines.

Agrobacterium-mediated transformation was done by floral dipping. The positive clones were inoculated into a YEP medium containing 50 mg/L rifampicin and 50 mg/L kanamycin, cultured at 28 °C for 12 h. Agrobacterium was collected, and transformation solution (500 mL containing 100 µL Silwet L-77, 25 g sucrose, 1.1 g MS) was adjusted to OD600 = 0.8–1.0. At full flowering stage, Arabidopsis was cut to remove the mature siliques, and the inflorescence was immersed in the transformation solution for about 60 s, sealed, and left overnight in the dark.

### 4.5. Identification of Mutants, Overexpression and Complementary Lines

T-DNA insertion mutants *pin3-1* and *pin3-2* were extracted separately; pistil-specific overexpression lines pSTK::PIN3-GUS (pBI101.3), genomic DNA of the complemented lines pPIN3::PIN3-GFP (pHB), identified with different primer pairs. Refer to Supplementary Table S3 for related primer combinations. 

### 4.6. Quantitative Real-time RT-PCR

Flowers of individual plants of independent transgenic lines of Col, mutants, over-expression (heterozygous) and complementation lines were collected, grounded in liquid nitrogen, and total RNA was extracted with TRIZOL (T9424, Sigma-Aldrich). FastKing RT Kit (with gDNase) kit (KR104, TIANGEN) was used for cDNA synthesis. qRT-PCR reaction system was prepared using SYBR Green Realtime PCR Master Mix (QPK-201, TOYOBO), and qRT-PCR was performed using a quantitative PCR instrument (Thermo Fisher & Quan Studio 3). The internal reference gene is *ANTIN*. All qRT-PCR primers used are listed in the Table S3.

## Supplementary Materials

The following are available online at https://www.mdpi.com/2223-7747/9/5/585/s1, Figure S1: Gene Expression Patterns of *PIN3*. Table S1: List of Arabidopsis accessions used in the study and their seed number data. Table S2: Details of potential candidate gene functions harboring SNPs affecting seed number per silique in *Arabidopsis*. Table S3: Primer sequences used in this paper.

## Abbreviations

GWAS	Genome-wide association analysis
SNS	Seed number per silique
SD	Seed density
STK	SEEDSTICK
CMM	Carpel margin meristem
SNP	Single nucleotide polymorphisms
OD	Ovule density
TGW	Thousand grain weight
