# Supplementary material for: Genome-Wide Association Analysis Identifies Candidate Genes Regulating Seed Number per Silique in Arabidopsis thaliana
Source: Plants (Basel). 2020 May 2;9(5):585. doi: 10.3390/plants9050585 (PMC7284809; doi:10.3390/plants9050585)
Supplement: Supplementary file 1 [file plants-09-00585-s001.zip › Supplementary files/Supplementary Figure S1.pdf]

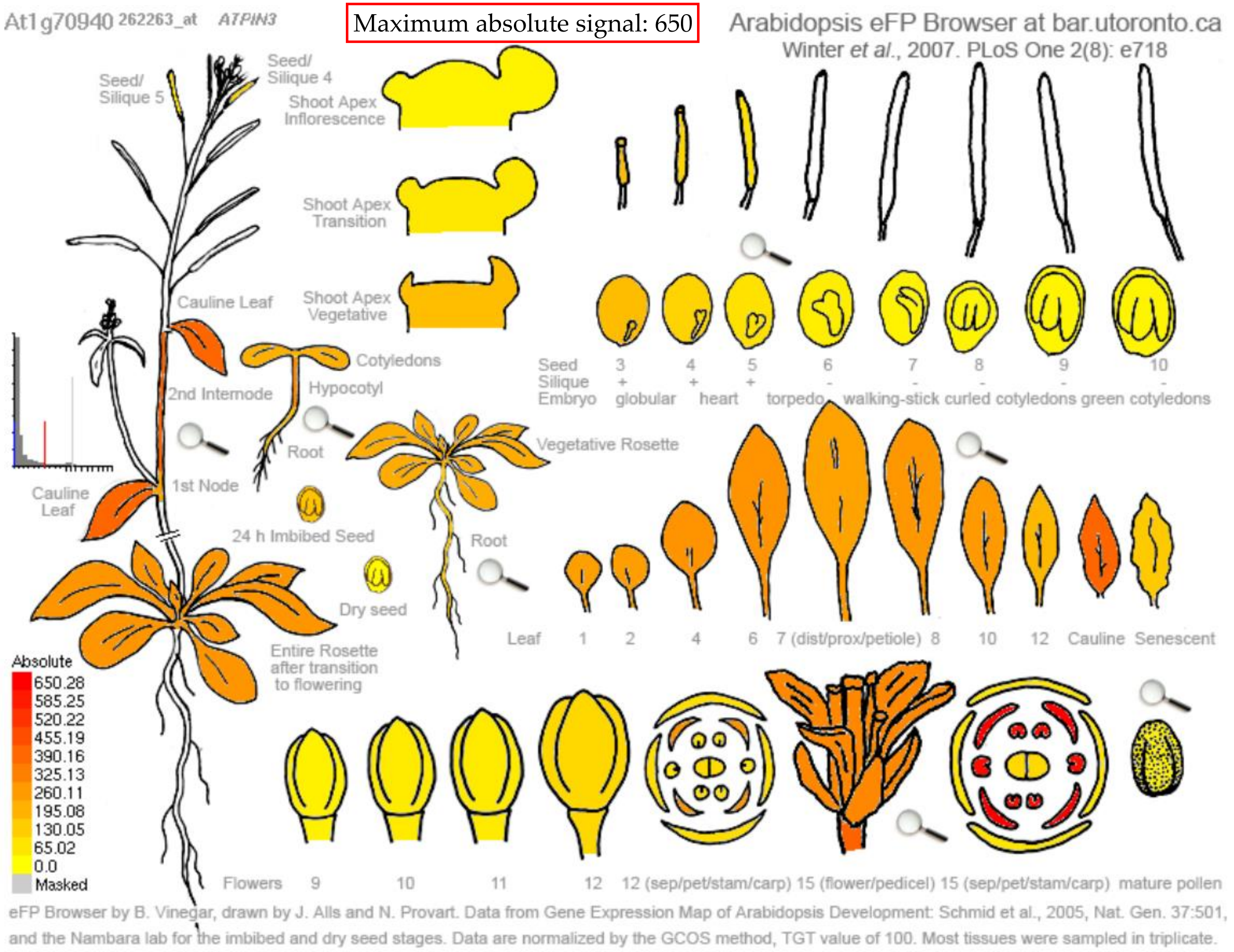

Figure S1. Gene Expression Patterns of *PIN3*.

Tissue expression pattern of *PIN3* gene from publicly available microarray datasets visualized by the Arabidopsis eFP Browser (http://bar.utoronto.ca/efp/cgi-bin/efpWeb.cgi?primaryGene=AT1G70940&modeInput=Absolute).
